# Microstructural Evolution and Phase Formation in 2nd-Generation Refractory-Based High Entropy Alloys

**DOI:** 10.3390/ma11020175

**Published:** 2018-01-23

**Authors:** Eyal Eshed, Natalya Larianovsky, Alexey Kovalevsky, Vladimir Popov, Igor Gorbachev, Vladimir Popov, Alexander Katz-Demyanetz

**Affiliations:** 1 Israel Institute of Metals, Haifa 3200003, Israel; eyale@trdf.technion.ac.il (E.E.); natalyal@trdf.technion.ac.il (N.L.); alekseyk@trdf.technion.ac.il (A.K.); vvp@trdf.technion.ac.il (V.P.J.); 2 Institute of Metals Physics Ural Branch of RAS, 620041 Ekaterinburg, Russia; gorbachev@imp.uran.ru (I.G.); vpopov@imp.uran.ru (V.P.)

**Keywords:** high-entropy, refractory, thermodynamics, solid solution, intermetallic phase

## Abstract

Refractory-based high entropy alloys (HEAs) of the 2nd-generation type are new intensively-studied materials with a high potential for structural high-temperature applications. This paper presents investigation results on microstructural evolution and phase formation in as-cast and subsequently heat-treated HEAs at various temperature-time regimes. Microstructural examination was performed by means of scanning electron microscopy (SEM) combined with the energy dispersive spectroscopy (EDS) mode of electron probe microanalysis (EPMA) and qualitative X-ray diffraction (XRD). The primary evolutionary trend observed was the tendency of Zr to gradually segregate as the temperature rises, while all the other elements eventually dissolve in the BCC solid solution phase once the onset of Laves phase complex decomposition is reached. The performed thermodynamic modelling was based on the Calculation of Phase Diagrams method (CALPHAD). The BCC A2 solid solution phase is predicted by the model to contain increasing amounts of Cr as the temperature rises, which is in perfect agreement with the actual results obtained by SEM. However, the model was not able to predict the existence of the Zr-rich phase or the tendency of Zr to segregate and form its own solid solution—most likely as a result of the Zr segregation trend not being an equilibrium phenomenon.

## 1. Introduction

High entropy alloys (HEAs) are, in relative terms, newcomers to the world of metallurgy. These alloys are composed of roughly equimolar proportions of metallic elements [1,2,3]. There are two major types of HEAs, each differing greatly from the other in its physical, chemical, and mechanical properties: the FCC HEAs comprise mostly of the late transition metals, while BCC HEAs comprise mostly of refractory metals [1,2,3,4]. Ideally, HEAs should possess a single disorderly solid solution phase, which lends them their high entropy characteristic [1].

It is common practice to classify the refractory element-based HEAs according to generations (1st, 2nd, and 3rd) based solely on their chronological formulation. The 1st-generation HEAs were the original compositions belonging to this family—Mo_25_Nb_25_Ta_25_W_25_ and Mo_20_Nb_20_Ta_20_V_20_W_20_, both are BCC and possess a single solid solution [4,5]. Owing to the resilience of HEAs at high temperatures, researchers most aspire to apply them in the aerospace industry (such as turbine engine blades). The one significant drawback to such an application is the high density and low corrosion resistance exhibited by the refractory elements, separately or alloyed [5].

The 2nd- and 3rd-generation HEAs categories relate to the desire of researchers to lower the density and impart corrosion resistance to these alloys. To that end, some of the heavier elements, such as W, Mo, and Ta, have been eliminated or reduced in quantity, while passivating elements that provide corrosion protection, such as Cr, Ti, Zr, and Al, have been introduced [6,7]. Plastic deformation involving changes and dislocations of the fine structure evolution occurring through rolling of Co-, Cr-, Ni-, Fe-, Mn-containing FCC-based cast HEA was studied by Klimova et al. [8]. The effect of yttria particle reinforcement on the microstructure and mechanical properties of CoCrNiFeMn-sintered HEA was investigated by Liu at al. [9]. A positive effect of ceramic reinforcement on high-temperature mechanical properties without a negative effect on tribological properties was established. Feng et al. [10] studied an effect of short-range order in FeCoNi(AlSi)_x_ HEAs on the magnetic properties and found a good coincidence between the experimental results and the results of a Monte Carlo simulation of the nearest neighbor distribution, as well as between the elastic modulus and magnetic moment.

Second-generation, which are the subject of this publication, usually comprise at least one of the core refractory elements, namely Nb, while incorporating elements which fall under the wider definition of refractory elements (elements which share only some of their attributes with the refractory element, such as a high melting point), such as Cr, Ti, V, Zr, and Hf. 

This alteration of composition in the 2nd-generation HEA results in the emergence of several phases, some of which having intermetallic nature, in the microstructure. From a thermodynamic standpoint, the stability of the multi-phase microstructure originates in a total high negative enthalpy of formation, which offsets the high entropy of the solid solution form, thereby enabling a highly-negative Gibbs free energy of formation for the multi-phase form. Having said that, at a sufficiently high temperature the entropy component of the free Gibbs energy ($-T\Delta S_{f}$) for the single solid solution form becomes so negative that it turns into the stable form. 

The purpose of this publication is to investigate the effect of the rise in temperatures over the microstructure of two 2nd generation HEAs—

Cr_20_Nb_20_Ti_20_V_20_Zr_20_

Cr_20_Mo_10_Nb_20_Ti_20_Ta_10_Zr_20_

## 2. Results

### 2.1. Cr_20_Nb_20_Ti_20_V_20_Zr_20_ Alloy

**Figure 1:**
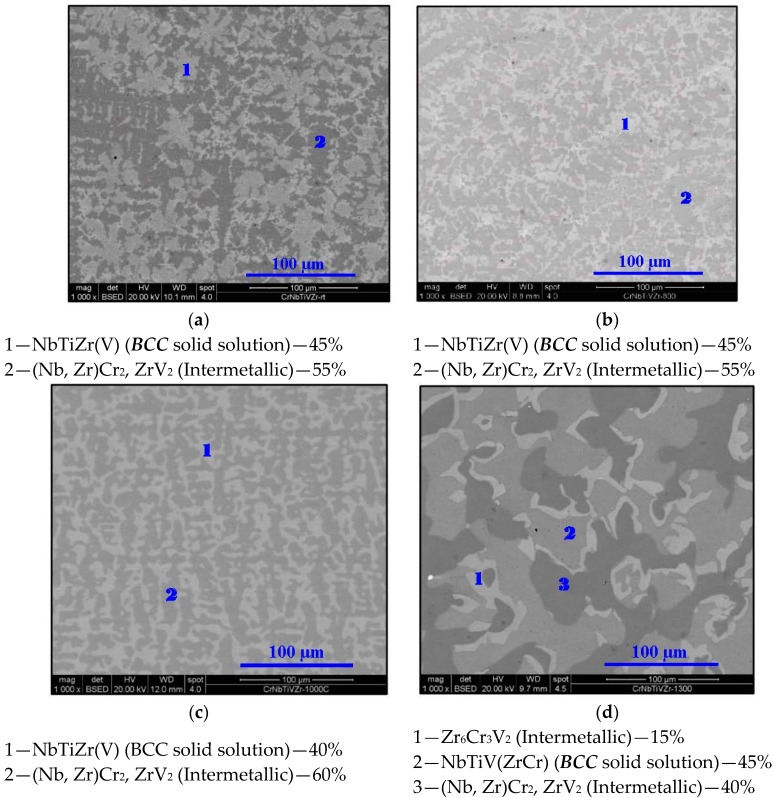
SEM micrographs of Cr_20_Nb_20_Ti_20_V_20_Zr_20_ at (**a**) 25 °C, (**b**) 800 °C, (**c**) 1000 °C, and (**d**) 1300 °C at 1000× magnification in BSE mode.

The following figures (Figure 1a–d) show the microstructure of the alloy at designated temperatures (i.e., the thermally-treated products) as captured by scanning electron microscopy (SEM). The associated phase compositions and fractions represent the approximated stoichiometry (based on energy dispersive spectroscopy (EDS) measurements) and volume percentages respectively. Parentheses in phase identification indicate substantial amounts of dissolved elements.

**Figure 2:**
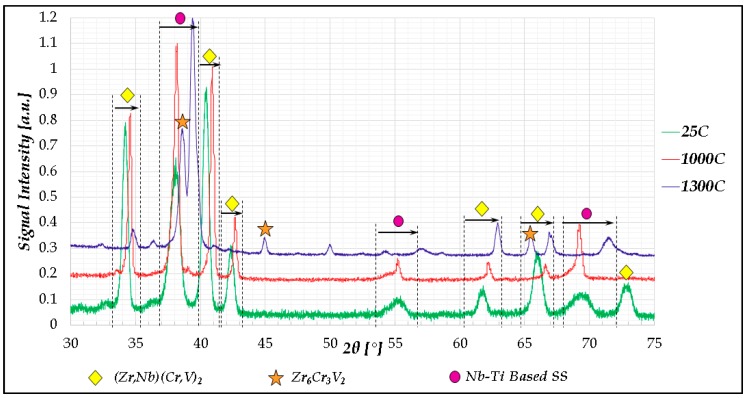
XRD traces of the Cr_20_Nb_20_Ti_20_V_20_Zr_20_ alloy at 25 °C, 1000 °C, and 1300 °C; the shifts in 2θ values of certain peaks as a result of the rising temperature are marked in arrows.

XRD was also utilized as a complementary characterization technique to verify the phase identification performed according to SEM. The following figure (Figure 2) presents the XRD spectra taken from cut portions of the thermally-treated products.

The data at 1600 °C for this alloy could not be clearly obtained due to a strong reaction between the Zr and the Al_2_O_3_ powder used in the experiment setting, resulting in a thick pure ZrO_2_ layer and incorporation of some Al in the Zr-depleted matrix. However, it may indicate that the Zr segregation process was finalized and the free Zr was able to react with Al_2_O_3_ as thermodynamics would otherwise dictate. Furthermore, no trace of the Zr rich phase was found. 

### 2.2. Cr_20_Mo_10_Nb_20_Ta_10_Ti_20_Zr_20_ Alloy

**Figure 3:**
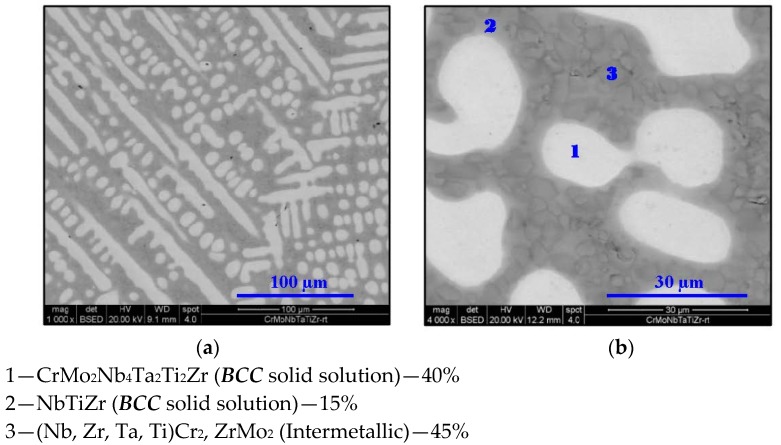
SEM micrograph of Cr_20_Mo_10_Nb_20_Ta_10_Ti_20_Zr_20_ at 25 °C at (**a**) 1000× and (**b**) 4000× magnifications in BSE mode.

**Figure 4:**
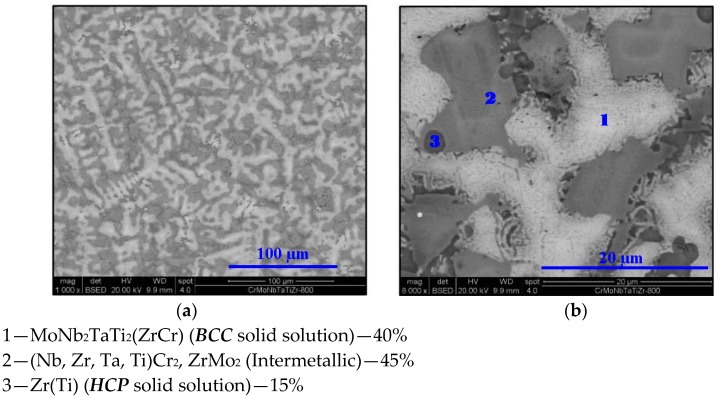
SEM micrograph of Cr_20_Mo_10_Nb_20_Ta_10_Ti_20_Zr_20_ at 800 °C at (**a**) 1000× and (**b**) 8000× magnifications in BSE mode.

**Figure 5:**
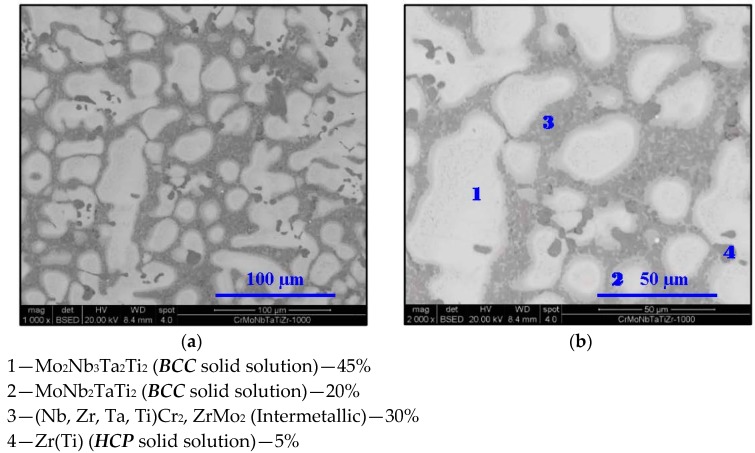
SEM micrograph of Cr_20_Mo_10_Nb_20_Ta_10_Ti_20_Zr_20_ at 1000 °C at (**a**) 1000× and (**b**) 2000× magnifications in BSE mode.

**Figure 6:**
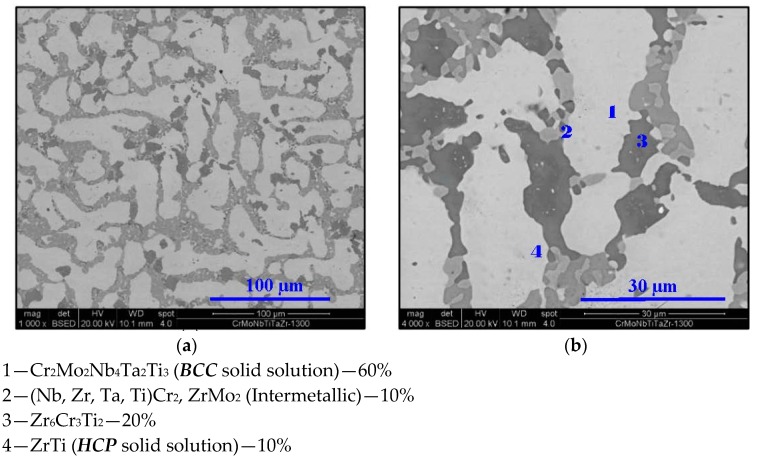
SEM micrograph of Cr_20_Mo_10_Nb_20_Ta_10_Ti_20_Zr_20_ at 1300 °C at (**a**) 1000× and (**b**) 4000× magnifications in BSE mode.

**Figure 7:**
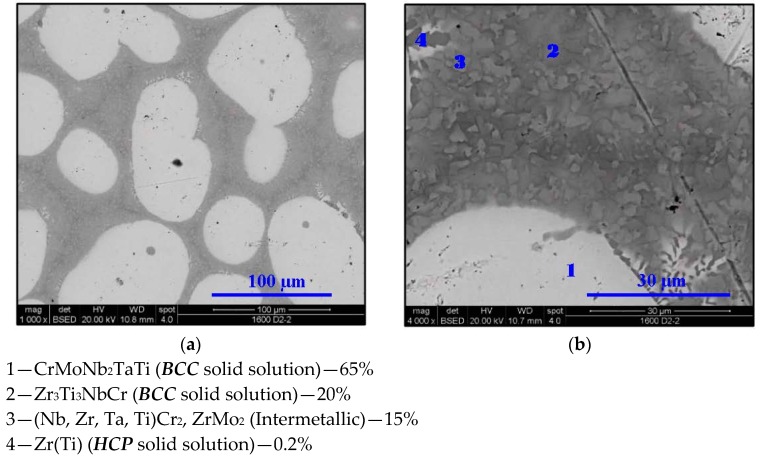
SEM micrograph of Cr_20_Mo_10_Nb_20_Ta_10_Ti_20_Zr_20_ at 1600 °C at (**a**) 1000× and (**b**) 4000× magnifications in BSE mode.

The following figures (Figure 3, Figure 4, Figure 5, Figure 6 and Figure 7) display the microstructure of this alloy at the designated temperatures as captured by SEM. The associated volume fractions of each phase are presented. Parentheses in the phase identification indicate substantial amounts of dissolved elements. 

**Figure 8:**
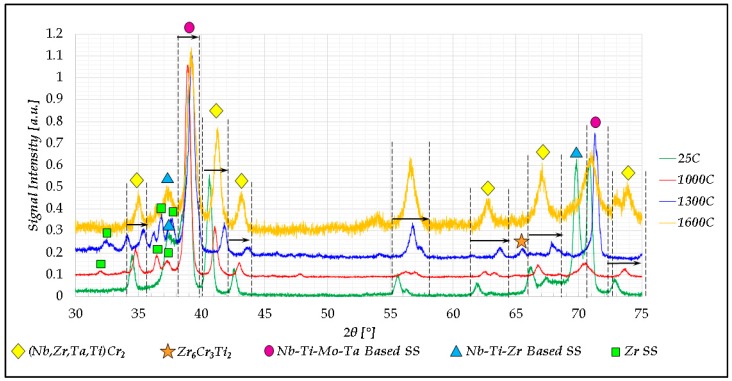
XRD traces of the Cr_20_Mo_10_Nb_20_Ta_10_Ti_20_Zr_20_ alloy at 25 °C, 1000 °C, 1300 °C, and 1600 °C; the shifts in 2θ values of certain peaks as a result of the rising temperature are marked in arrows.

The following figure (Figure 8) presents the XRD spectra taken from cut portions of the thermally-treated products of the Cr_20_Mo_10_Nb_20_ Ta_10_Ti_20_Zr_20_ alloy.

## 3. Discussion

**Figure 9:**
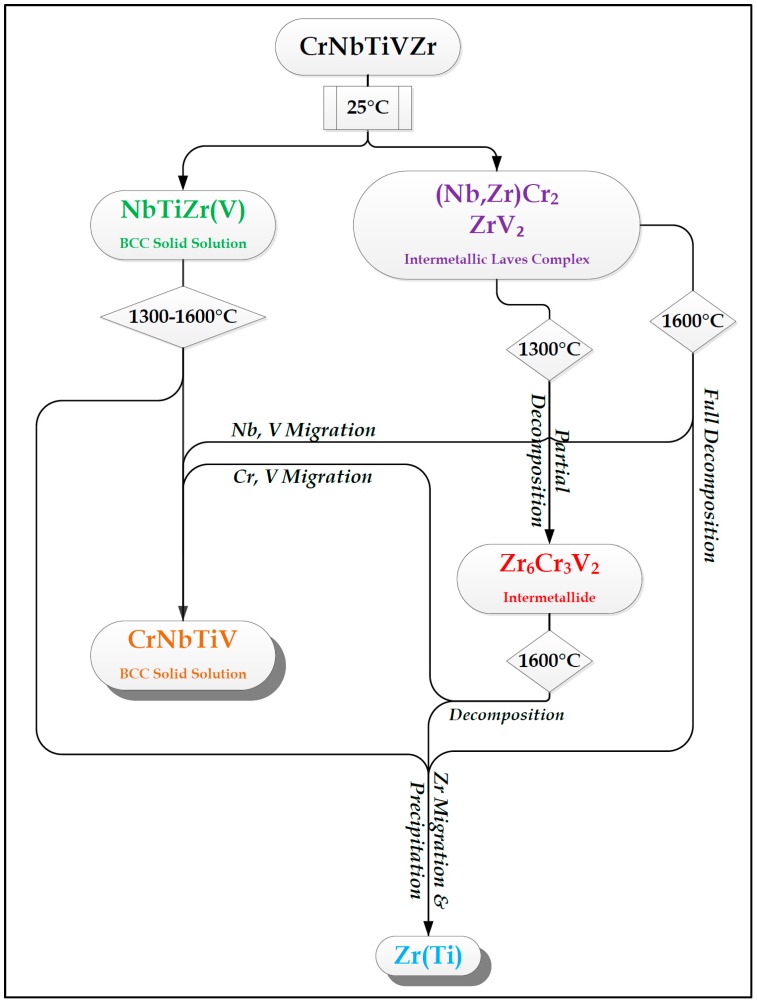
Flowchart depicting the evolution of phases as the temperature rises in the Cr_20_Nb_20_Ti_20_V_20_Zr_20_ alloy. Diffusion processes occurring at 1600 °C are tentative.

**Figure 10:**
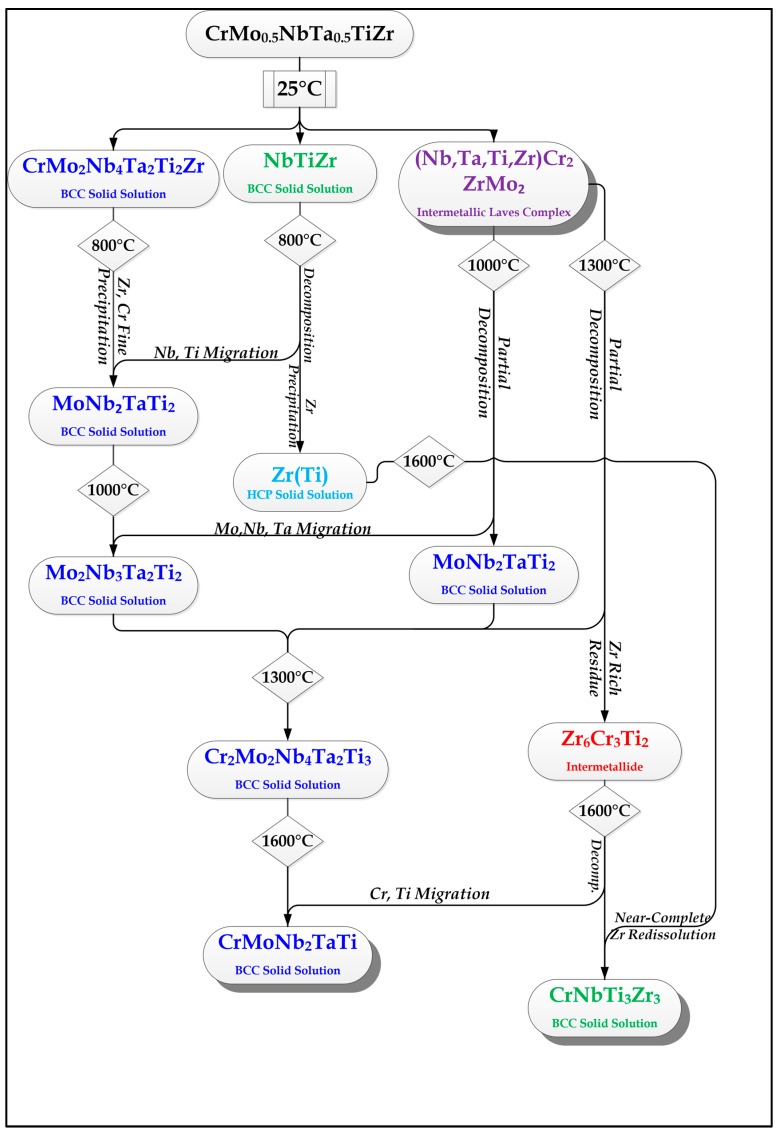
Flowchart depicting the evolution of phases as the temperature rises in the Cr_20_Mo_10_Nb_20_Ta_10_Ti_20_Zr_20_ alloy.

**Figure 11:**
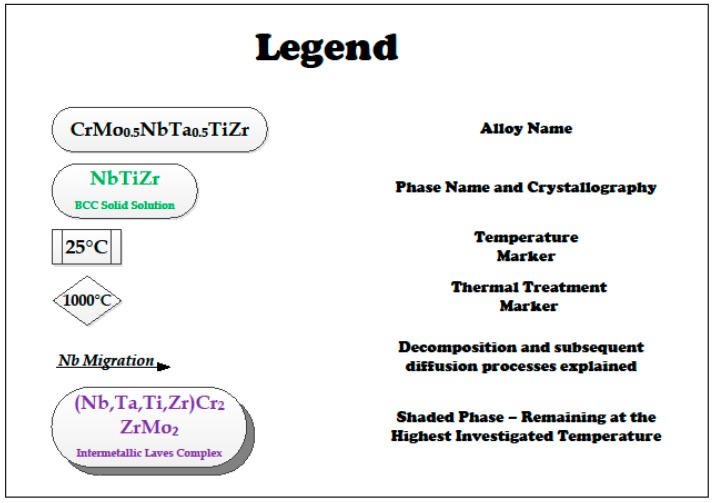
Legend for the flowcharts in Figure 9 and Figure 10.

The phase identification performed at each examined temperature for both alloys, based on the EDS measurements, is supported and reinforced by the qualitative XRD analysis. The following Figure 9 and Figure 10 summarize the main diffusion-lead processes leading up to the formation, decomposition, and dissolution of phases and species at every examined temperature for both alloys. The legend for the figures is shown in Figure 11.

The main evolutionary trend seen in the flow charts is the tendency of Zr to segregate as the temperature rises, while Cr begins to dissolve in the BCC solid solution phase/s when the Laves complex begins to decompose.

The segregation of Zr is not linear with temperature, and sometimes it is done through the formation of HCP Zr(Ti) solid solution phase islands while at other times it is done by capturing other elements, which immediately turn the Zr-Ti-based phase from HCP to BCC. It is possible that the decomposition of the Laves complex is what drives this segregation or separation. Since Cr dissolves in in the majority BCC phase once the Laves phase decomposition point is reached, the Zr is forced to immediately separate into its own domains and may take other elements with it in the process. The binary Zr-Cr can serve as a testament to this behavior. 

## 4. Thermodynamic Modelling 

Thermodynamic calculations of phase equilibrium in the Cr_20_Nb_20_Ti_20_V_20_Zr_20_ alloy at 800, 1000, 1300, and 1600 °C have been performed based on the CALPHAD-method using the algorithm determined in [11]. This algorithm was tested previously in thermodynamic calculations for a number of multicomponent systems (see for example [12,13,14,15,16]), and appropriate results were obtained. In this method, the calculation is performed by finding an alloy phase composition corresponding to the global minimum of its Gibbs free energy. Mathematically, the Gibbs free energy (*G*) of a system is a function of many variables, such as component concentrations in phases and mole fractions of phases. Thus, the search for the *G* function’s minimum was performed in the presence of non-linear limitations in the form of equalities and inequalities, i.e., a general problem of nonlinear programming was solved. To find phase compositions corresponding to the global minimum of the Gibbs free energy a special procedure of choosing starting points described in [11] was used. 

In calculations used for the description of condensed phases, a sublattice model of Hillert-Staffonsson [17] was used, generalized by Sundman and Agren for a case of several sublattices [18]. According to [18], an expression for a phase Gibbs free energy is written as a function of mole fractions of every *i*-th element in the *s*-th sublattice $Y_{i}^{s}$:

(1) $$G^{\prime }=\underset{I0}{\sum }\underset{IO}{\prod }{\left(Y\right)}^{0}G_{I0}^{hf}+RT\overset{l}{\underset{s=1}{\sum }}a_{s}\overset{N}{\underset{i=1}{\sum }}(Y_{i}^{s}lnY_{i}^{s}+Y_{Va}^{s}lnY_{Va}^{s}){+}^{mag}G^{f}+\underset{Z>0}{\sum }\underset{IZ}{\sum }\underset{IZ}{\prod }\left(Y\right)L_{IZ}$$

Here, *R* is the universal gas constant, *T* is temperature, *Va* denotes vacancies, *a_s_* is a number of sites in sublattice *s* per one mole of formula units of a phase, $Y_{i}^{s}$ is a mole fraction of component *i* in sublattice *s* of phase *f*. Parameter GI0f0 denotes Gibbs free energy of one mole of formula units of a compound with the same crystal structure as phase *f*, corresponding to an element of array *I*0 determining one element for every sublattice; ∏I0(Y)GI0hf0 denotes the product of the corresponding elements of matrix ‖Y‖; *I*1 denotes the array determining such variants of atoms distribution in sublattices where one sublattice contains atoms of two elements, whereas the rest contains atoms of only one element; and $\prod_{I1}\left(Y\right)$ corresponds to the product of the appropriate elements of matrix ‖Y‖. Array *I*1 is referred to as the first order array contrary to the array of 0th order. Arrays of higher orders *IZ* correspond to various combinations of higher numbers of elements from different sublattices. Parameters *L_IZ_* characterize the interaction energy of components of the corresponding element of array *IZ*.

For a liquid phase a model of regular solution was used, according to which its molar Gibbs energy is expressed as follows:

(2) $$G^{L}=\underset{i}{\sum }X_{i}^{L0}G_{i}^{L}+RT\overset{N}{\underset{i=1}{\sum }}X_{i}^{L}lnX_{i}^{L}+\underset{Z}{\sum }\underset{IZ}{\sum }\underset{IZ}{\prod }\left(X\right)L_{IZ}^{L}$$

where $X_{i}^{L}$ is the mole fraction of the *i*th component in a liquid phase; GiL0 is the molar Gibbs energy of pure component *i* in a liquid state; and $L_{IZ}^{L}$ are the solution parameters.

Nowadays, the CALPHAD method is most widely used for thermodynamic description of concentration dependences of thermodynamic properties of phases. A distinguishing feature of the CALPHAD method is that the thermodynamic description of high order systems is based on those of lower order systems. To ensure mutual consistency of different systems of higher orders, similar descriptions of lower order systems are used.

As a rule, for the thermodynamic description of multicomponent systems the procedure is as follows: Firstly, the thermodynamic descriptions of binary systems are summed. Then, they are supplemented with interaction parameters of third, fourth and higher orders taking into account specific features of three-component systems, four-component systems, etc. However, the available experimental data and thermodynamic descriptions are often not enough for doing so. In this case, the lacking parameters are assumed equal to zero. 

The thermodynamic description of the Cr-Nb-Ti-V-Zr system was performed this way. According to the available literature data, there are sufficient descriptions of sub-systems belonging to this system. For the thermodynamic description of the Cr-Nb-Ti-V-Zr system, we preferred the data, which is based on the SGTE database for pure elements [19], which are maximally formally consistent with each other and take into account up-to-date experimental information. There are thermodynamic descriptions for all binary systems involved. For the systems Cr-Nb, Cr-Zr, and Nb-Zr the thermodynamic data was taken from [20], for the Ti-Zr, V-Zr, and Ti-V systems, the source was [21]; for Cr-Ti and Cr-V the source was [22]; for Nb-Ti we used the findings of [23]; and for Nb-V the data was based on publication [24]. Parameters of third-order interactions are available only for three systems: Cr-Ti-Zr [10], Ti-V-Zr [21], and Cr-Nb-Ti [22], and the data on fourth-order parameters are absent in the literature. The lack of data on considerable number of thermodynamic parameters could surely affect the calculation accuracy, and that is why the obtained thermodynamic description and corresponding calculations should be considered only as the first approach. 

The formation possibility of the following phases was taken into account in calculations: liquid phase, solid solutions with BBC and HCP crystal lattices, and Laves phases C14, C15, and C36. 

Table 1 summarizes the phases making up the microstructure at each investigated temperature in equilibrium:

The general increasing trend of the solid solution content as the temperature rises, which is predicted by the model, is in line with the actual findings of the scanning electron microscopy analyses; however, the absolute values do not correlate. 

Furthermore, The BCC A2 solid solution phase is predicted by the model to contain increasing amounts of Cr as the temperature rises, which is in agreement with the actual results.

The tendency of Zr to gradually segregate, forming a Zr-rich phase and its own solid solution (with some dissolved Ti) is probably a non-equilibrium process resulting from the “frozen” state of the as-cast products, which solidified relatively quickly after arc melting. In other words, the thermal treatments only serve as a means to relieve the stresses that exist in the as-cast products and enable some diffusion, to a degree the specific temperature permits. These diffusion processes can only bring the microstructure closer to equilibrium state but cannot fully reach it, especially at low temperatures.

Additionally, the Laves complex phase is predicted to consist mostly of NbCr_2_ with negligible V amounts, which is not the actual case (it consists mostly of ZrCr_2_ and ZrV_2_).

## 5. Materials and Methods 

All materials used for the research have been supplied by Holland Moran Ltd. (Yahud, Israel) The first stage of the experiments consisted of alloying. Both alloys were prepared in Materials Research Furnaces Inc. vacuum arc melting furnace produced in Allenstown (NH, USA) model ABJ-900-1-VD-LL-02/GG-VAC-BF, while the nominal mixtures of the corresponding elements were used. All elements had a purity of 99.5%. Arc melting was conducted on a water-cooled copper plate. High-purity molten titanium was used as a getter for residual oxygen, nitrogen, and hydrogen. 

**Figure 12:**
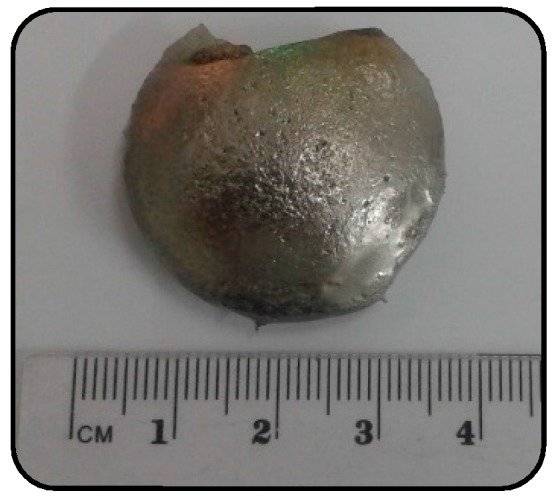
Exemplary cast button of HEAs.

To achieve a homogeneous distribution of elements in the alloys, each alloy was re-melted four times, flipped before each melting run, and kept in a molten state for 5 min during each run. This resulted in a button-shaped product shown in Figure 12.

The second stage in the experimental procedure was the thermal treatments. The cast buttons were cut and annealed at various temperatures, before being quenched in water. The following procedures were conducted in each temperature. At 800 °C, cut portions of the products were treated for 24 h using ‘Sen Pak’ steel envelopes as oxidation protection. At 1000 °C and 1300 °C, the samples were annealed for 24 h in quartz ampoules and at 1600 °C bars were annealed for 5 h in an alumina crucible filled with condensed high-purity alumina powder and covered by an alumina lid.

Analyses of microstructure in each temperature was performed using FEI Inspect scanning electron microscope (SEM) (FEI, Brno, Czech Republic), equipped with energy dispersive spectroscopy (EDS) detector. An accelerating voltage of 20 kV was used; the working distance was 10 mm. A Rigaku SmartLab 9-KW X-ray diffractometer (XRD) (Rigaku, Tokyo, Japan) was employed for qualitative X-rays diffraction analysis. The scattering range was 30–75°; the scan increment was 0.01°. To define the volume fraction of each phase, optical microscope Olympus SC-30 (Olympus, Tokyo, Japan) equipped for pixel-based image analysis using the ‘Stream Essentials’ (Olympus, Tokyo, Japan) software was used. The images analyzed by this method were obtained by SEM.

## 6. Conclusions

1. In high-entropy alloys based on Zr and BCC elements, such as Cr, Ti, and Nb (the first two falling under the wider definition of refractory elements and Nb being a refractory element), a unique phenomenon of gradual Zr segregation as the temperature rises can be observed. This phenomenon is apparently related to the contrasting crystal structures of the HCP Zr solid solution and the BCC Cr-Nb based solid solution. It is more than likely that this phenomenon is not in equilibrium since Zr is expected to have a BCC structure at high temperatures as well.
  Some Ti is also incorporated in the secluded Zr solid solution regions and, to a small degree, it behaves in the same manner, being an HCP element itself.
2. The Zr segregation is initiated by the onset of Laves phase decomposition at around 1000 °C, which leaves behind a Zr-rich phase. The other elements are then incorporated in the increasingly Zr-depleted BCC solid solution phase, which gradually grows in volume as the temperature rises further.
3. The thermodynamic simulation successfully predicts the general trend of increasing volume percentage of the Cr-inclusive solid solution phase, as well as the increasing Cr incorporation in it, as a function of temperature (around 1000 °C and higher), however, Zr segregation is not accounted for, due to the fact that it may not be an equilibrium phenomenon. The Nb-Ti-Zr based solid solution phase appearing at low to moderate temperatures in both alloys forms as a result of the relatively rapid cooling associated with arc melting and is not likely to be the equilibrium solid solution phase predicted at those temperatures by the model.

## Figures and Tables

**Table 1:** Model prediction of chemical composition of every phase in equilibrium at the designated temperatures.

Temperature (°C)	Phase	Weight Fraction (%)	Chemical Composition (at%)				
			Ti	V	Cr	Zr	Nb
**800 °C**	BCC_A2	**72.68**	23.86	28.29	2.04	26.52	19.29
	Laves_C15	27.32	10.74	0.14	63.02	4.39	21.71
**1000 °C**	BCC_A2	**74.88**	23.24	27.30	4.73	24.90	19.83
	Laves_C15	25.12	11.21	0.19	61.46	6.68	20.46
**1300 °C**	BCC_A2	**86.14**	21.34	23.42	13.23	21.69	20.32
	Laves_C15	13.86	12.25	0.26	59.13	10.22	18.14
**1600 °C**	BCC_A2	**100.00**	20.00	20.00	20.00	20.00	20.00
